# Revisiting the Nordic long-term care model for older people—still equal?

**DOI:** 10.1007/s10433-022-00703-4

**Published:** 2022-05-03

**Authors:** Tine Rostgaard, Frode Jacobsen, Teppo Kröger, Elin Peterson

**Affiliations:** 1 Roskilde University, Roskilde, Denmark; 2 Stockholm University, Stockholm, Sweden; 3 Western Norway University of Applied Science, Bergen, Norway; 4 University of Jyväskylä, Jyvaskyla, Finland

**Keywords:** Long-term care, Nordic countries, Retrenchment, Privatisation, Informalisation

## Abstract

With the extensive long-term care services for older people, the Nordic countries have been labelled ‘caring states’ as reported (Leira, Welfare state and working mothers: the Scandinavian experience, Cambridge University Press, Cambridge, 1992). The emphasis on services and not cash benefits ensures the Nordics a central place in the public service model (Anttonen and Sipilä, J Eur Soc Policy 6:87–100, 1996). The main feature of this ideal model is public social care services, such as home care and residential care services, which can cover the need for personal and medical care, as well as assistance with household chores. These services are provided within a formally and professionally based long-term care system, where the main responsibility for the organization, provision and financing of care traditionally lays with the public sector. According to the principle of universalism (in: Antonnen et al. (eds), Welfare state, universalism and diversity, Elgar, Cheltenham, 2013), access to benefits such as home care and residential care is based on citizenship and need, not contributions nor merit. Also, care services should be made available for all and generally be used by all, with no stigma associated. Vabø and Szebehely (in: Anttonen (ed), Welfare State, universalism and diversity, Edward Elgar Publishing, London, 2012)) further argue that the Nordic service universalism is more than merely issues of eligibility and accessibility, in that it also encompasses whether services are attractive, affordable and flexible in order to meet a diversity of needs and preferences. However, recent decades have seen a continuous tendency towards prioritization of care for the most frail, contributing to unmet need, informalization of care and privatization in the use of topping up with market-based services. These changes have raised questions about increasing inequalities within Nordic long-term care systems. We investigate in the article what effect changes have for equality across social class and gender, for users and informal carers. The article is based on analysis of comparable national and international statistics and a review of national research literature and policy documents.

## Introduction

Equality is a key characteristic for the Nordic countries, sometimes presented as the greatest legacy of the twentieth century in this region (Kvist et al. 2011). With the shared ‘passion for equality’ (Marklund 1988), the Nordics have organized the approach to welfare around this value, including how social services such as long-term care for older people in the form of home care and residential care homes are distributed in the population. Accordingly, social services are generally organized on the basis of universalism and citizenship, not on membership or merit. The ideal is therefore that any inequality in social background should be evened out to ensure an equitable allocation of care services. Long-term care can be provided by the state, market, volunteers or family members and unique for the Nordic countries is the high degree of involvement of the state, and especially of the local municipalities. To ensure that policy decisions regarding services are taken as close to the individual as possible and responding to local needs, the municipalities carry the main responsibility for organisation, financing and provision of services. Often, the generosity of the Nordic long-term care system has been highlighted; based on a comparison of 14 European countries, Anttonen and Sipilä (1996) in an often-cited article concluded that it was in this region that the highest proportion of older people 65 + received residential care or home help. In the welfare literature, this encompassing approach to social services has earned the Nordic countries the label of ‘caring states’ (Leira 1992) and their particular model of welfare has been named ‘the public service model’ (Anttonen and Sipilä 1996). Over time, the Nordic countries have established what is by international standards, a most generous model of long-term care for older people, which is ideally used by all, without social stigma, thereby ensuring equality in access to services across the social class divide and equity in regard to the distribution of services.

As a second and equally important equality feature inherent in the Nordic public service model is also the assumption of gender equality and the facilitation of the dual earner-dual carer model (Ellingsaeter and Leira 2006; Wright et al. 2009). With the high involvement of the (local) state in long-term care services, the family has traditionally played a more supplementary role unlike many other European countries. Therefore, quite paradoxically, the Nordic countries are often portrayed as having the highest numbers of informal caregivers but also the lowest numbers of intensive caregivers, as informal care giving may not be time-intensive and often can be performed outside work hours (Verbakel 2018). Women are less tied up in providing informal care and can instead participate on the labour market, catering for a gender inclusive and participatory model of citizenship where ideally both men and women can act as citizen-earners/carers as well as carers/earners.

However, as is the case in other European countries with ageing populations, changing demographics have put pressure on the municipalities for finding more efficient and alternative approaches to care provision, often in competition with increasing demands also from other policy areas, such as child-care and assistance for persons with disability. Earlier investigations of the Nordics have identified a number of major developments going in the direction of retrenchment, informalization and privatization (Rostgaard and Szebehely 2012; Szebehely and Meagher 2018). Increasingly, former generosity in LTC services is replaced with prioritization of service provision, targeting those sick and frail older people with the greatest care needs. Instead, older people (and their families) top-up public service delivery by purchasing supplementary market-based services, often by means of tax rebates. Such privatization tendency may have implications for social class inequality. Changes not only relate to who and how many receive care. There are indications of policy changes also in the content of the care that is provided and essentially what is to be part of public responsibility for care provision and what is intended for the family (or market) to provide. This may have consequences for the division of care work between the public services and informal carers, with clear gender implications in that women (also in the Nordic countries) are traditionally more often involved in informal care giving.

The aim of this article is therefore to take stock of the developments in the Nordic long-term care model over the last two decades and investigate the consequences regarding equality, for older people but also for those caring for them. The research questions investigated are whether policy changes of retrenchment, informalization and privatization are similar across the countries or unique to some? Are policy changes in the main long-term care services, home care and residential care, piecemeal and unintentional or deliberate by actual reform? And what are the consequences regarding equality across gender and social class? We investigate these questions across four Nordic countries (Denmark, Finland, Norway and Sweden).

## Methods and data

In order to conduct a comparative study of the changes in institutional features of national LTC systems and the implication for equality, we first identified the relevant analytical categories to be studied. These were identified a priori by the authors following common discussions and by drawing on the existing literature and our expertise in the field. For instance, our discussions on gaps in the comparative approach to social care services revealed that comparative studies often do not pay attention to disparities within countries (Kröger 2011).

In accordance, the author group identified the following analytical categories as relevant for the study of equality in LTC: changes over the last two decades in 1. Overall policy goals behind LTC, 2. Regulation and organization, 3. Formal and informal division of care work and 4. Variation within countries. In the analysis, we assessed these four analytical categories according to the implication for users as well as for informal carers with regard to inequality across gender and social class. The analysis proceeded in the following way: we each investigated these categories in the national setting, based on statistics and available research literature and policy documents, and engaged in common discussions about how to interpret the changes and their impact on equality. Therefore, the empirical data consist of national research and public policy documents as well as comparable statistics^1^ which has been assessed in regard to implication of changes for social and gender inequality.

### Equality and long-term care in a Nordic lens

A traditional approach to equality in the welfare literature has been income equality and the re-distributional effect of cash transfers. This reflects that welfare state research has for many generations concentrated on cash benefits in the male breadwinner welfare state. Here, the particular equalizing effect of the Nordic universal income protection programmes has been highlighted (see, e.g. Korpi and Palme 1988). Later generations of welfare researchers have identified that also, with regard to social services, the Nordic model had inherent traits that made it stand out in the production of equal opportunities and equitable outcomes. In fact, Lewis and Daly (2000) have claimed that only in the Nordic region is it possible to identify a cluster of countries that have a similar approach to care rights and obligations. Contrary to many other countries, there is in the Nordic countries no formal obligation nor strong norms for the family to care for older parents.

A traditional explanation for this is the generous provision of social services which has unburdened the family and in particular has managed to relieve women of some of their care responsibilities for older parents. Relative to other European countries, the Nordics seem to have achieved a modicum of gender equality in informal caregiving with little gender care gap (da Roit et al. 2015). In these countries, women and men are considered equally employable and often working full-time. As a result, the Nordic model has been successful in facilitating gender equality by integrating women and mothers into the labour market and political system (Pascal and Lewis, 2004). The Nordic model has also been highlighted for the universal approach to the provision of care services which ensures equality in access to as well as equity in use of social services, such as long-term care. As Anttonen (2002) points out, universalism is an ideal type, somewhat ‘out of reach’. However, she identifies a number of key equalising elements of the universalistic approach to care services in the Nordic countries. These include identical rights to tax-financed social services for all citizens; services are intended to be of similar quality throughout the country; services are defined by compulsory legislation; and services are designed for the entire population, who have equal access and the majority of whom are users. Service universalism means that care provision is based on needs assessment and not selectivism, which could favour particular groups in society (Anttonen 2002).

In other words, the particular Nordic approach to care aims to ensure that inequalities across socio-demographic and socio-economic status are not translated into inequity in the use of services. Instead, as an ideal, services are awarded irrespective of income and place of residence. This is not to say that the service provision is uniform. Another ideal trait of the Nordic service universalism is that services are flexible and individualized, catering for a heterogeneous population with different cultural preferences and lifestyles. Equally important for ensuring equality in access and equity in use of services is that care services are affordable, generously available and of high quality (Vabø and Szebehely 2012).

However, unlike cash benefit such as sickness benefit or unemployment benefits where assessment of eligibility is relatively straight forward (being ill/losing one’s job), long-term care services are generally allocated according to an assessment of individual need with less clear-cut eligibility criteria. There is therefore no national, legislative right to care services in the Nordic countries but a right to individual needs assessment and a formal decision at the local level. It has been argued that this undermines the principle of universalism, which at best is only ‘weak’ universalism (Kröger 2003; Kröger et al. 2003; Szebehely and Meagher 2018). Given the large autonomy of the municipalities, some variation is to be expected but nevertheless poses a continuous challenge for determining which factors are to be considered sources of ‘fair’ variation and which are to be considered ‘unfair’.

The person’s level of frailty, measured as physical or cognitive limitation, is an obvious and fair factor for allocating care services at the local level, and however, other factors are less clear, and with implications for equality across both gender and the social class divide. An example of such an unclear issue is whether accessibility to long-term care should depend on household composition and family situation. There may not be a formal obligation for family members to provide care. However, also in the Nordic countries, spouses and adult children are important sources of informal care (Jakobsson et al. 2013; Ulmanen and Szebehely 2015) and a classical conundrum is whether the municipal assessment of need should take into account such available resources. Regardless, even access to informal care is seldom evenly distributed; older people with higher socio-economic condition tend also to have greater household size, and closer distance to children (Weyers et al. 2008). This suggests that higher income groups have easier access to informal care (Ilinca et al. 2017), although they may not need to be dependent on informal care, as they have better resources to purchase market-based care.

The family situation may also in more indirect ways impact inequalities in the use of LTC services. Having a partner or adult child may be influential in the presentation of the needs situation and the negotiation with the municipality over which and how much care should be allocated (Larsson and Silverstein 2004, see also the article by Erlandsson et al. in this special issue of EJoA). Other factors of variation may present themselves as more straightforward. Across different care regimes, income-related differences as well as differences related to the person’s education or place of residence are generally considered to be illegitimate if they are decisive for how care services are allocated (Ilinca et al. 2017).

## Results

Based on our thematic analysis of national research and policy documents as well as comparison of available statistics, we have identified four changes in the LTC systems and policies that have had effect for social and gender inequality: 1. Unequal and non-equitable distribution of services, 2. Retrenchment and prioritization of resources, 3. Informalisation and finally 4. Privatisation. In the following sections, we present the trends and assess their inequality impact.

### Unequal and non-equitable distribution of services

Regardless of the aim of producing equality in access to and equity in use of service in the Nordic region, this does not mean that service provision is identical within the countries. Given the high level of local autonomy, there are traditionally substantial local differences in the service levels (Kröger 1997; Rausch 2007; Trydegård and Thorslund 2010). This suggests that there is not a uniform ‘welfare state’ in the Nordic countries but rather a large number of ‘welfare municipalities’ that differ substantially from each other, not least in service levels, i.e. the proportion of a given population group receiving services as well as the quality of such services.

The local variation in LTC service levels is often historically embedded and cannot be explained by the political constellation in a given municipality. That is to say, earlier studies have not been able to conclude that municipalities with, e.g. a social-democratic orientation, have traditionally favoured long-term care higher than municipalities with a liberal-bourgeois orientation (e.g. Jensen and Lolle 2015). Rather it has been, in particular, demographic factors which seem to have driven variation in service levels, such as the proportion of 80 + and of single older households, as well as the median income in the municipality (Davey et al. 2006; Savla et al. 2008). In other words, the welfare literature has tended to establish previously that it is local variation in need that to a large degree defines the municipal variation in service provision and expenditure across the Nordic municipalities, not political ideology. Therefore, Davey et al. (2006) in a previous study stated that the distribution of services was perhaps unequal across municipalities, but nevertheless seemed equitable at the time.^2^

More recent studies, however, indicate that a change may have occurred which have created less equitable outcomes, and which positions frail older people differently, according to which municipality they live in. In the case of Norway, newer evidence suggests that variation in need cannot fully explain the local variation in LTC service levels. A recent study utilizing national statistical data from Statistics Norway (KOSTRA data)^3^ and the Directorate of Health and Care Services (KPR/IPLOS)^4^ find clear priority differences, where, for example, municipalities prioritize differently as to general coverage of LTC services (between 32 and 76% coverage in 2018 for population 80 +). In addition, there seems to be municipal variation in how they balance between institution-based and home-based care services and in the quality of services provided (Førland et al. 2020). Also, in Finland, there are large municipal variations in expenditure and in the total coverage of LTC services (between 15 and 40% for 75 + population) which cannot be explained by variation of need (THL 2018; Tupala et al. 2020). In Denmark, home help hours have fallen disproportionally in municipalities outside the capital area in the period 2008–2019 (not controlling for variation in need) (Ruge and Houlberg 2020). Overall—and in contrast to the findings of Davies et al.—this suggests that geographical distribution of LTC services may be both unequal and inequitable but mainly due to lack of resources than due to particular political orientation.

### Retrenchment and prioritization of resources

There may be indication of more unequal LTC service distribution within the municipalities, but across the municipalities, there are also clear signs of overall retrenchment and prioritization of resources. In fact, the development in Sweden suggests that the overall tendency to cut back services has resulted in less variation across municipalities as they all have followed the same trend of retrenchment (Szebehely and Trydegård 2018).

Rather than this trend towards retrenchment and prioritization being an outcome of healthy ageing or explicit national or local policy, it seems to be a clear prioritization of resources locally, aimed at covering needs mainly for older people with multiple care needs (see, e.g. Rostgaard and Matthiessen 2019; Rostgaard et al. 2020). These tendencies seem to be related to how need is assessed, and services allocated locally, but nevertheless have derived implications for social class and gender equality.

These overall trends towards retrenchment and prioritization are evident when looking at the coverage of LTC services which has diminished gradually over time in all the Nordic countries. The universalistic LTC model in the Nordic countries was in the 1990s characterized by reaching a large proportion of the older population 65 + , if combining home and residential care with cash for care allowances, e.g. 27% in Denmark and 21% in Sweden. This distinguished the Nordic countries from countries with more residual LTC models (such as France, Germany, Spain, Italy) where coverage levels were rarely above 10% of the older population at this time (Ranzi and Pavolini 2015).

**Fig. 1:**
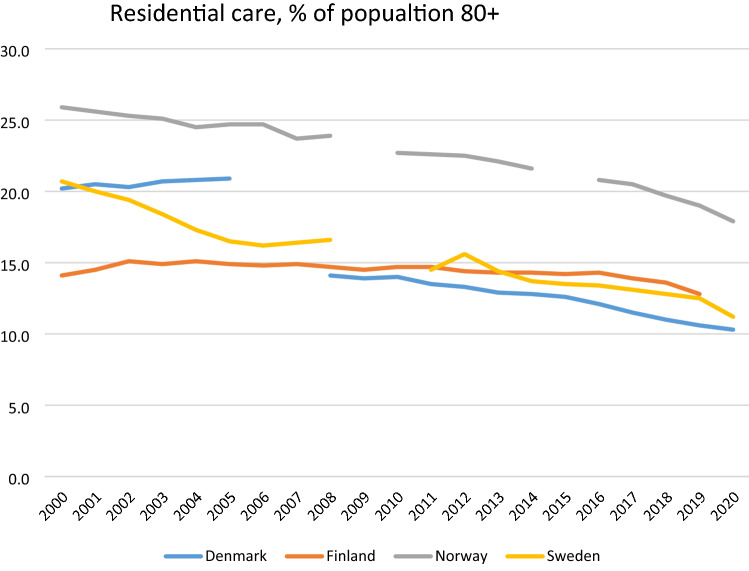
Users of residential care services, % of 80 + , 2000-most recent year. *Sources*: Residential care—https://www.nordicstatistics.org/social-integration-and-income/day-care/ SOCI22: People aged 65 + living in institutions or service housing, by unit, age, time and reporting country, for the period 2000–16. This is supplemented with national data for the period 2017–2020. Note that Danish data before 2004 and after 2008 are not comparable due to a change in the how to account for residential care

**Fig. 2:**
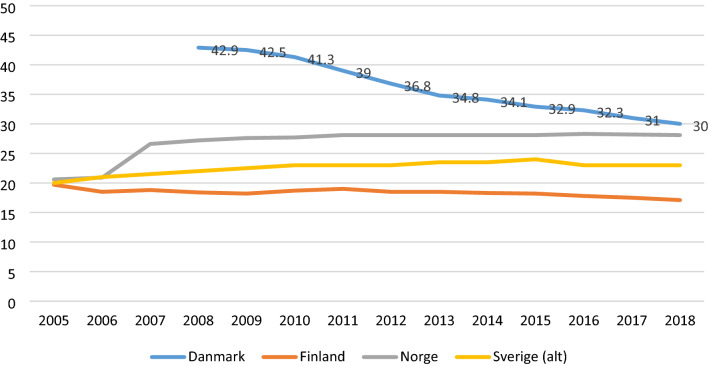
Users of home care, % of 80 + , 2000-most recent year. *Sources*: OECD Health Care Statistics (xxxx) and Socialstyrelsen (2021). *Note* In Denmark and Finland, this includes only those who receive regular home care services, not intermediate services

As illustrated in Figs. 1 and 2, and now looking at 80 + , coverage levels for home care and residential care have steadily decreased over the years in the Nordic countries. The Nordic countries vary in what services have been primarily affected by the changes in LTC in the last two decades. In Sweden, Norway and Finland, it is mainly access to residential care that has been reduced, whereas also coverage of home care in Denmark has declined.

Focussing first on residential care, all the countries have for years advocated the ageing-in-place policy, why home care has priority over residential care. However, with an ageing population and subsequently an increase in dementia among the oldest-old, it could be expected that the use of residential care would increase. As Fig. 1 illustrates this is not the case, as coverage of home care has stayed more or less the same. Rather, the changes in recent years suggest an adjustment of the role of residential care —and as is visible in Fig. 2, this is not compensated by home care.

The decline in residential care has been particularly sharp in Sweden in the last two decades. Between 2000 and 2015, every fourth residential care bed disappeared (SOU, 2017). Access among older adults aged 80 + has gone from 20 per cent in 2000 to 11 per cent in 2020 (Fig. 1) and the increase in home care services has not been able to cover up for the retrenchment in residential care (Szebehely 2020). Waiting time for a place in residential care was in 2020 64 days, an increase from 51 days in 2008. In approx. half of the cases, the municipalities quote lack of resources as the cause (Socialstyrelsen 2021). The development towards fewer available places also reflects a turn towards dementia-friendly environments, choice and personalization. In both Norway and Finland, traditional residential care, often within a hospital-like setting with shared facilities such as toilets and baths, has been reduced (or in the case of Finland nearly vanished) (Kröger 2019). Residential care is now more often single-occupancy, with smaller wards frequently with 8–12 residents, with a consumerist approach in that there is a selection of services that needs to be paid for separately.^5^ In both countries, less costly alternative arrangements such as assistive living and supportive housing have grown. In Denmark, there is a break in data, but also here coverage rates have gone down since 2008. As an illustration of increasing discrepancy between supply and demand, the average waiting time for a bed in residential care has here increased from 22 to 31 days in the period 2010–2019 in Denmark (Sundheds- og Ældreministeriet 2020).

Recent changes have in Denmark affected, especially access to home care. In 2008, as many as 43% of the 80 + age group in Denmark received home care, personal care and/or cleaning services; today this is 30% (Fig. 2). Denmark has in this way followed the path of the other Nordic countries, which had already in the 1990s changed the provision of home care, from ‘reaching the many with some home care hours’ to ‘reaching the few with intensive hours’. E.g. in Finland, coverage was dramatically reduced by 40% in the beginning of the 1990s (Kröger and Leinonen 2012). However, as coverage fell in Denmark, hours have not increased: average number of hours have for the 80 + declined from 3.9 h in 2008 to 3,3 h in 2019 (Ruge and Houlberg 2020). The reason seems to be an implicit municipal policy of recalibrating home care services towards covering mainly personal care. Since 2008, cleaning services have been cut back severely in Denmark, from a visit weekly to sometimes only monthly, making the assistance with household chores more symbolic than real.

Figure 2 illustrates that the coverage of home care either declines (as in Denmark) or more or less stays the same (Finland, Norway and Sweden). In other words, in none of the countries do the changes in home care compensate for the decline in residential care. An often-used explanation is that ageing populations are more healthy and fit and have less need for care. This development may have been supported by the introduction of reablement in home care, an intervention focussed on training of daily activities. In Denmark and in many municipalities in Norway, this intervention is now the first choice before the municipalities offer conventional home care services. Ideally, these interventions could improve functional ability and therefore reduce the need for home care, although the evidence is still scarce (Rostgaard et al. forthcoming). However, a recent Danish study investigates this thesis by comparing developments in need and the coverage of home care since the national introduction of reablement. The study concludes that the reduction in home care coverage in Denmark is not the result of more healthy ageing, but that it has instead resulted in an increase in self-reported unmet need, with implications for quality of life (Rostgaard and Matthiessen 2019, Rostgaard et al. 2020; for Finland, see Kröger et al 2019).

The targeting of the most frail in all the Nordic countries implies that access to home help is prioritized for those with highest needs (and in Denmark and Norway only after the possibility to offer an alternative reablement intervention has been exhausted). E.g. in Sweden, home care users have more and more complex care needs, and many require home nursing as well (Socialstyrelsen 2020). Overall, a smaller proportion of older adults receives more intensive care, and the focus of home care has changed from domestic tasks (e.g. cleaning) to assistance with personal care (e.g. bathing) and tasks with nursing/medical components. Weaker recognition of social and emotional needs also leads to a higher risk of loneliness and unmet needs for the user. Residents in nursing homes in the same way are more frail before they are admitted to residential care.

### Informalisation

Parallel to the changes in home care and residential care, there has been a trend of informalisation of care in the Nordic countries. In contrast to other countries, adult children are not legally obliged to provide or pay for the care for their aged parents, but informal help performed by adult children and other family members is increasing—in actual involvement but also as a result of changing policy expectations as to what is the role of the family. The shift in focus of home care towards personal care and nursing/medical tasks requires families to be more involved in providing help and support related to household work and social and emotional support. This is despite that there is no indication in changes in norms about family obligations, which in a European perspective have traditionally placed much less weight on the role of the family in the provision of care for older people (Verbakel 2018).

The increasing demand for informal caring is a consequence of the combination of retrenchment and a change in how the role of the family is addressed in public discourse, and in some cases, also how care allowances paid to the carers or the cared-for, is presented as an alternative to services. Such as in Finland, where the use of care allowances for informal care has become increasingly common and families are widely assumed to participate in the care for older relatives at home (Kalliomaa-Puha 2017; Kröger 2019). Also, in residential care, family members are expected to contribute; a practice that backfired when visits where prohibited due to the COVID-19 pandemic. In Norway, the family is portrayed as key in the ‘co-creation of care’ in White papers and other national policy papers, thus appealing to a popular governance concept, and presenting informal caring as a way to ensure empowerment and better care. Here, family members’ involvement in the care for older people is deemed necessary to ensure sustainability of services in the future (Christensen 2018). A small but growing number of family carers receive a salary for care (omsorgslønn), granted and paid by the local authorities (Statistics Norway 2018). In these two countries, informalization of care seems to be an explicit policy priority, and as in the case of Norway, sugar-coated with the reference to personalization and better quality of care.

In contrast, increasing reliance on the family seems in Denmark and Sweden to be a derived effect of the pragmatic cut-backs in municipal home care, rather than the result of explicit national or local policy reform, and in both countries, care allowances play only a marginal role. Even so, the trend of informalisation has been even more evident in Sweden than in the other Nordic countries (Szebehely and Meagher 2018). As such, increased family care has been an unintended consequence of the decline in care services in Sweden (Ulmanen 2017). Similarly, in Denmark, the policy towards involving family and friends in care for older people is officially unchanged, and care allowances for informal care are not commonly used. Nevertheless, in the context of reduced home care coverage, Danish families are likely to experience more pressure to provide informal help and support (Rostgaard and Matthieseen 2019, Rostgaard et al. 2020).

Informalisation processes tend to reinforce class and gender inequalities (Szebehely and Meagher 2018). Due to their longer life expectancy, more women than men need care and therefore increasingly need to rely on informal care. Women as partners and adult daughters are also more likely to be involved in providing informal care and those who are active in the labour market, may feel squeezed and experience problems of work-life balance (Riedel and Krauss 2011). Also, family care is most extended among older people with lower levels of education (i.e. lower incomes) who more often do not have the means to purchase market-based care as an alternative to publicly provided care (Rostgaard and Szebehely 2012; Rostgaard and Matthiessen 2019; Mathew Puthenparambil et al. 2017; Ulmanen and Szebehely 2015).

### Privatisation

The need to find other sources of care such as market-based care is an indirect result of the increasing targeting in the Nordic countries but nevertheless supported by policies of care allowances and tax rebates and the focus on personal care. Following from this, the use of private out-of-pocket domestic services, generally provided by for-profit companies, has become more common among older people (Moberg 2017). In Finland, for example, services such as cleaning and shopping are predominantly provided by the market, which generates needs for additional help and support for those who cannot afford to purchase private care (Mathew Puthenparambil et al. 2017).

Also, Finland, Denmark and Sweden have introduced tax deductions on domestic services. These tax rebates are not specifically directed to the older population but are often used by this age group as well (Szebehely and Meagher 2013). For example, in Sweden, the age cohort in which the tax deduction is used the most is the one between 85 and 95 years (Brodin and Andersson 2017; Erlandsson et al. 2013). Older people can use tax rebates to top-up their needs-assessed services, but they can also use them to contract private services as an alternative to needs assessed home care. In Sweden and Finland, the tax rebates can be used for both personal care and domestic services, whereas it can be used only for specific household tasks such as cleaning in Denmark. Further, in Sweden and Finland, only private for-profit providers are allowed to offer tax-deductible domestic services. Given that user fees in home care are both income-related and dependent on the number of hours of help, privately purchased domestic services can be cheaper than home care, for older people with higher incomes and smaller care needs (Ulmanen and Szebehely 2015). Norway has not adopted any similar tax rebate, but topping-up is becoming more common. These services mainly consist of for-profit provision of practical help for home-dwelling older adults. Norway offers no tax deductions for practical help, but has sustained a system of co-payment, where the user pays the same amount regardless of public or private service provider (Norwegian Government 2014).

Topping-up constitutes a parallel market-based care system where personal economic resources are essential for meeting need. A public care system which increasingly refers to or relies on the user to purchase care in order to top-up services therefore has obvious implications for social class inequality (Hjelmar and Rostgaard 2020). Not surprisingly, privately purchased help is generally more common among older people with higher incomes (Mathew Puthenparambil et al. 2017; Rostgaard and Matthiessen 2019).

## Conclusion

Across the Nordic countries, there are continuous trends over time for retrenchment in LTC services with clear implications for equality as a trademark of universalism. This development in LTC has been identified already some years ago, concluding that weak universalism has become weaker but mostly so in Finland and Sweden (Kröger 2003; Kröger et al. 2003; Szebehely and Meagher 2018). Recent development suggests that the trend of de-universalisation is now also present in Denmark and Norway—and with implications for equality across gender and social class in all countries. In contrast to many other European countries, where universalistic characteristics have been introduced in reforms of LTC services only since the turn of the century (Leon et al. 2014), the Nordic model seems to be under long-term re-construction but nevertheless continuing the direction of more restricted universalism (Ranci and Pavolini 2015).

The changes most often seem to be piecemeal and incremental and not the result of larger policy reforms or political ideologies, neither at the central nor local government level. Only in Finland and to some degree Norway, does the development seem to be driven by explicit policy changes underlining the role of informal carers and supported by cash for care. The retrenchment is evident from the declining coverage of services which is not explained by more healthy ageing in the older population, and first and foremost driven by a need for prioritizing resources. As a result, LTC services are increasingly targeted at the most frail. More specifically, it means that the requirement for entry into residential care has become more strict, fewer receive home help and home help hours may be reduced. In all countries, cleaning was earlier considered part of home care services but now focus is more and more on personal and medical care needs to a degree—as in the case of Denmark—where the small number of hours of home help allocated for cleaning becomes symbolic and devoid of meaning.

Revisiting Anttonen’s key characteristics of universalism (Anttonen 2002), there are still identical rights to tax-financed social services, in the sense that there is a right to be assessed for need and those services which are provided by the public sector are still mainly tax-financed. (Although affordability is an issue in residential care.) However, targeting of resources means that services are no longer used broadly across the population but increasingly so by the (targeted) few. It becomes a matter of definition whether targeting also violates the principle of universalism that prescribes equal access, a hallmark of equality. Retrenchment at first sight seems to be ‘democratically’ applied across, for instance, local municipalities, i.e. everyone is ‘equal’ in receiving less, as in the case of Sweden. However, there are indications of local variation in use of services which cannot be explained by need. This suggests that new trends of geographical inequality may be at play in the Nordic region which again may enforce social inequality between urban/rural and rich/poor areas. Equally so, tendencies of informalization and privatization may amplify inequalities across gender and social class. Leaving the informal carers to pick up where the public sector left has gendered implications in that women often constitute the majority of those in need for care and of those providing informal care. Informalisation also creates inequalities between those who have and those who do not have a well-functioning relationship with family members nearby, while privatization and marketisation have obvious implications with regard to social class and the ability to pay for topping-up services.

Overall, with regard to LTC services and the adjustment to new demographic realities, the Nordic model seems to have lost its breath somewhat and can no longer guarantee that inequalities across socio-demographic and socio-economic status are not translated into inequity in the use of services. The changes not only have implications on the individual level, for older people and their family members, but also more overall suggest a recalibration of the Nordic model. It seems not to be deliberate but nevertheless is effectual.

## Footnotes

1 Cross-country comparison of LTC systems constitutes a general and continuous problem, despite recent advancements in the OECD Health Statistics. Even in the Nordic countries, where LTC systems are relatively similar and individual data systematically registered, the comparison of such statistics poses a methodological problem, especially over time. For instance, the countries take a different approach to what is included in the concept of residential care and often there are breaks in the data. Accordingly, the comparison of statistics has been made cautiously and we point to the methodological limitations where relevant.

2 Davey et al. make the reservation that they in the study only had simple data on coverage of services and no data on intrinsic quality of the home help or number of hours provided.

3 https://www.ssb.no/en/offentlig-sektor/kostra.

4 https://www.helsedirektoratet.no/tema/statistikk-registre-og-rapporter/helsedata-og-helseregistre/iplos-registeret.

5 A similar process took place in Denmark and Sweden already in the late 1980s and early 1990s.
