# Correction to: Revisiting the Nordic long-term care model for older people—still equal?

**DOI:** 10.1007/s10433-022-00712-3

**Published:** 2022-06-16

**Authors:** Tine Rostgaard, Frode Jacobsen, Teppo Kröger, Elin Peterson

**Affiliations:** 1 Roskilde University, Roskilde, Denmark; 2 Stockholm University, Stockholm, Sweden; 3 Western Norway University of Applied Science, Bergen, Norway; 4 University of Jyväskylä, Jyvaskyla, Finland

## Correction to: European Journal of Ageing (2022) 19:201–210 https://doi.org/10.1007/s10433-022-00703-4

In the original publication of the article, the title is corrected from “Revisiting the Nordic long-term care model for older people—still equal? Special issue EJA, edited by Fritzell, Jylhä and Rostgaard” to “Revisiting the Nordic long-term care model for older people—still equal?”.

The original article has been updated.

